# Repeated hypergravity training facilitates vestibular compensation associated with dorsal root ganglion remodeling

**DOI:** 10.1016/j.jphyss.2026.100100

**Published:** 2026-09-08

**Authors:** Yuta Masuda, Kakeru Shimizu, Marina Khatun, Sajjad Hossain, Risa Shimane, Nanaka Tameike, Yukari Takeda, Chikara Abe

**Affiliations:** a Department of Physiology, University of Fukui Faculty of Medical Sciences, Fukui, Japan; b Department of Biochemistry and Molecular Biology, Faculty of Life Science, Mawlana Bhashani Science and Technology University, Bangladesh

**Keywords:** Vestibular compensation, Hypergravity, Dorsal root ganglion, Somatosensory plasticity, Vestibular lesion

## Abstract

Vestibular compensation relies on sensory reweighting, but whether repeated hypergravity training (HT) facilitates recovery after bilateral vestibular lesion (BVL) remains unclear. We investigated the effects of HT on motor function and dorsal root ganglion (DRG) transcriptomes in mice following BVL. Mice underwent sham surgery or BVL and were assigned to either HT (2 G, 60 min/day for 10 days) or no HT (NHT). Body weight, righting reflex, and rotarod performance were evaluated, and RNA sequencing of L5 DRGs was performed on day 7. HT did not improve body weight loss or vestibular-dependent righting reflex deficits. However, HT resulted in significantly better rotarod performance on Day 7 compared with NHT. RNA sequencing identified 397 candidate genes, with enrichment of muscle-related and myelination-related biological processes. These findings suggest that repeated HT facilitates vestibular compensation by promoting transcriptional changes in DRGs and enhancing peripheral somatosensory plasticity rather than restoring vestibular function itself.

## Introduction

The vestibular system exhibits remarkable plasticity in response to altered gravitational environments and vestibular injury [1], [2], [3], [4]. We have previously demonstrated that prolonged exposure to hypergravity induces functional plasticity in the vestibular system [5], [6], [7]. Specifically, chronic hypergravity exposure attenuates the gain of the vestibulo-cardiovascular reflex, likely because sustained hypergravity reduces dynamic vestibular stimulation by limiting head movements and decreasing fluctuations in otolith afferent activity [5], [7], [8]. Importantly, this reduction in reflex gain can be restored by vestibular electrical stimulation, indicating that vestibular plasticity is reversible through appropriate sensory stimulation [9].

Hypergravity exposure also impairs vestibular-dependent motor behaviors, including the righting reflex, suggesting that reduced vestibular input compromises not only autonomic regulation but also motor function [6]. However, recovery from vestibular dysfunction is thought to involve sensory reweighting, whereby somatosensory and visual information compensate for the loss of vestibular input. Although proprioceptive inputs from the limbs are considered essential for vestibular compensation [1], whether enhancing somatosensory input facilitates functional recovery after bilateral vestibular loss remains largely unknown.

Based on these observations, we hypothesized that repeated hypergravity training (HT), which augments somatosensory input, would enhance somatosensory-driven compensation and improve motor function after bilateral vestibular lesion (BVL). To test this hypothesis, we compared two behavioral tasks with different sensory requirements: the righting reflex, which primarily depends on vestibular and visual inputs, and the rotarod test, which additionally requires coordinated limb movement and proprioceptive feedback [10], [11], [12]. Furthermore, because the dorsal root ganglia (DRGs) constitute the primary relay of peripheral somatosensory information, we performed RNA sequencing of L5 DRGs to determine whether HT induces transcriptional remodeling associated with improved motor performance.

## Methods

### Animals

Male C57BL/6 J mice (8 weeks old, n = 21) were obtained from Sankyo Labo Service Corporation (Tokyo, Japan). Mice were housed under controlled environmental conditions (22–24 °C, 12-h light/12-h dark cycle) with free access to standard laboratory chow and water. Animals were randomly assigned to experimental groups. All experimental procedures were conducted in accordance with the Guiding Principles for the Care and Use of Animals in the Field of Physiological Science and were approved by the Animal Research Committee of the University of Fukui (Approval No. R08012).

### Bilateral vestibular lesion

BVL were induced under isoflurane anesthesia. After removal of the tympanic membrane and auditory ossicles, labyrinthine fluid was aspirated through the oval window. PERIODON (Neo Dental International, Japan), containing dibucaine and paraformaldehyde, was then applied through the oval window to induce vestibular lesions by protein denaturation of the vestibular sensory organs [6]. Successful lesions were confirmed by characteristic postural abnormalities immediately after surgery and persistent impairment of the righting reflex. Sham-operated mice underwent removal of the tympanic membrane only, while the auditory ossicles remained intact. All surgical procedures were performed under aseptic conditions.

### Hypergravity training

Hypergravity exposure was performed using a gondola-type centrifuge with a 50-cm arm radius (Takei Scientific Instruments Co., Ltd., Japan). Centrifugation at 55 rpm generated a hypergravity environment of 2 G. The 2-G condition was selected based on our previous studies demonstrating that exposure to 2 G induces reproducible functional and neural adaptations in the vestibular system in mice [6], [13]. BVL mice were randomly assigned to either the HT group or the No HT (NHT) group. HT was initiated on the day after BVL surgery (Day 1), and mice were exposed to 2 G for 60 min once daily for 10 consecutive days at the beginning of the light phase. NHT and sham-operated (1 G) mice remained under normal gravity throughout the study.

### Righting Reflex

Vestibular-dependent motor function was evaluated using the righting reflex test. Mice were dropped upside down from a height of 50 cm, and the time and distance required to regain a normal ventral-down orientation were recorded using a high-speed camera (60 fps; iPhone 14, Apple, Cupertino, CA, USA). Video analysis was performed using Kinovea software (version 2025.1.1). Three trials were performed for each mouse, and the mean values were used for statistical analysis.

### Rotarod experiment

Motor coordination was assessed using a rotarod apparatus (Model 47600, Bio Research Center, Nagoya, Japan). Mice were placed on the rotating rod facing opposite to the direction of rotation. The rotation speed was gradually accelerated from 2 to 40 rpm over 2 min, and the latency to fall was recorded.

### RNA sequencing and bioinformatic analysis

On day 7 after BVL, L5 DRGs were collected from HT and NHT mice at least 2 h after completion of the behavioral assessments (n = 3 per group). Representative photographs of the DRGs were obtained before the DRGs were immediately frozen in liquid nitrogen, and stored at −80 °C until RNA extraction. RNA sequencing libraries were prepared using the SMARTer Stranded Total RNA-Seq Kit v2 – Pico Input Mammalian (Takara Bio, Mountain View, CA, USA) according to the manufacturer's instructions. Libraries were sequenced on a NextSeq 500 platform (Illumina, San Diego, CA, USA) to generate paired-end reads.

FASTQ files were imported into CLC Main Workbench (version 24.0.1; Qiagen). Reads were mapped to the mouse reference genome (mm10), and gene expression levels were quantified as total counts and reads per kilobase of transcript per million mapped reads (RPKM). All library preparation, sequencing, and primary bioinformatic analyses were performed at the Tsukuba i-Laboratory (Tsukuba, Japan).

### Experimental protocol

**Fig. 1:**
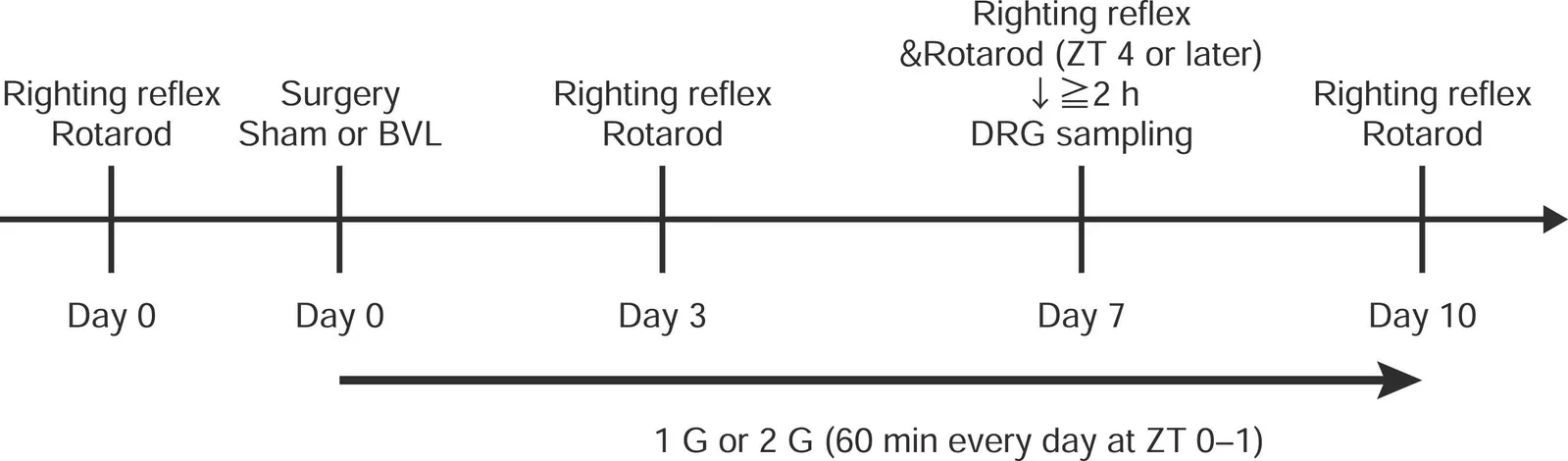
Experimental design. Experimental timeline of the study.

The experimental timeline is illustrated in Fig. 1. Body weight, righting reflex, and rotarod performance were evaluated before surgery (Day 0) and on days 3, 7, and 10 after BVL or sham surgery. HT was initiated on the day after surgery (Day 1) and was performed for 60 min at the beginning of the light phase. On behavioral assessment days, the righting reflex and rotarod tests were conducted at ZT4 or later, at least 3 h after completion of the daily HT session. The righting reflex test was performed first, followed by the rotarod test. On day 7, mice were anesthetized with ketamine (120 mg/kg) and xylazine (12 mg/kg), and L5 DRGs were harvested from the HT and NHT groups for RNA sequencing at least 2 h after completion of the behavioral assessments. At the time of DRG collection on Day 7, unilateral gastrocnemius and soleus muscles were also collected and weighed (n = 3 per group).

### Statistical Analysis

All statistical analyses were performed using GraphPad Prism version 11 (GraphPad Software, San Diego, CA, USA). Data were assessed for normality using the Shapiro–Wilk normality test, and homogeneity of variance was evaluated using the Brown–Forsythe test. Statistical comparisons were performed using an unpaired *t*-test or two-way repeated-measures ANOVA, as appropriate. For repeated-measures analyses, the Geisser–Greenhouse correction was applied when appropriate. Tukey’s multiple-comparison tests were used for post hoc comparisons, as appropriate. Data are presented as mean ± SEM. Statistical significance was defined as *P* < 0.05.

For RNA sequencing analysis, candidate genes were identified using a raw *P* value < 0.05 and an absolute difference in normalized expression values ≥ 20. Gene Ontology (GO) enrichment analysis was performed using these candidate genes, and GO terms with a Benjamini-Hochberg-adjusted P value < 0.05 were considered significantly enriched. Gene Ontology enrichment analysis was performed by Tsukuba i-Laboratory (Tsukuba, Japan).

## Results

### Repeated HT improves motor coordination without restoring vestibular-dependent reflexes

**Fig. 2:**
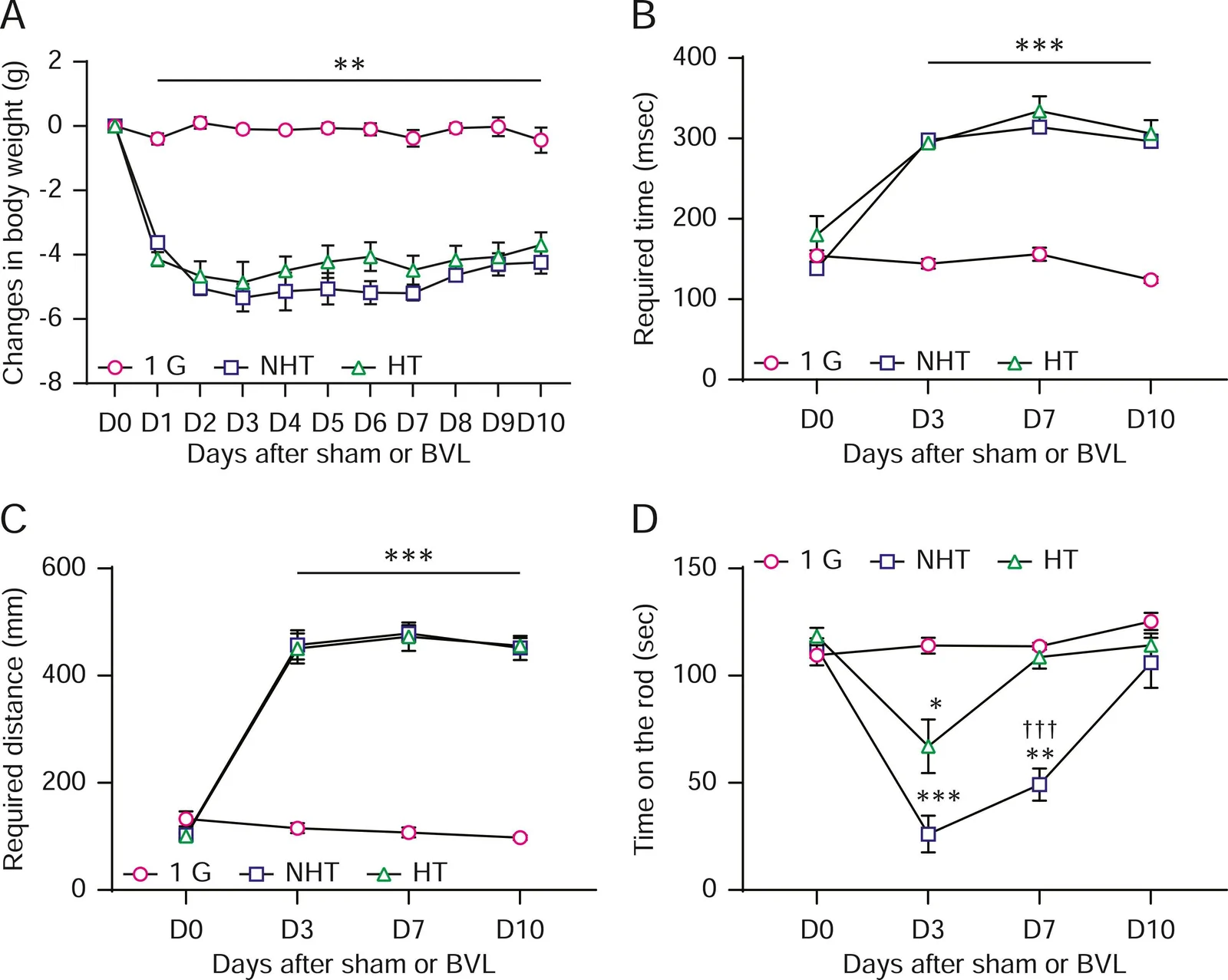
Repeated hypergravity training selectively improves motor coordination after bilateral vestibular lesion. (A) Changes in body weight following sham operation (1 G) or bilateral vestibular lesion (BVL) (n = 5 in each group). Data are presented as mean ± SEM. Statistics: two-way repeated-measures ANOVA with Geisser-Greenhouse correction followed by Tukey’s multiple comparisons test; group × time interaction [F (3.636, 21.82) = 8.510, *P* = 0.0004]. **P < 0.01 vs 1 G. (B) Righting reflex time measured before surgery (Day 0) and on days 3, 7, and 10 after sham or BVL surgery. Data are presented as mean ± SEM. Statistics: two-way repeated-measures ANOVA with Geisser-Greenhouse correction followed by Tukey’s multiple comparisons test; group × time interaction [F (3.823, 22.94) = 14.59, *P* < 0.0001]. ***P < 0.001 vs 1 G. (C) Righting distance before surgery (Day 0) and on days 3, 7, and 10 after sham or BVL surgery. Data are presented as mean ± SEM. Statistics: two-way ANOVA repeated-measures with Geisser-Greenhouse correction followed by Tukey’s multiple comparisons test; group × time interaction [F (4.591, 27.54) = 35.95, *P* < 0.0001]. ***P < 0.001 vs 1 G. (D) Rotarod performance before surgery (Day 0) and on days 3, 7, and 10 after sham or BVL surgery. Data are presented as mean ± SEM. Statistics: two-way repeated-measures ANOVA with Geisser-Greenhouse correction followed by Tukey’s multiple comparisons test; group × time interaction [F (4.523, 27.14) = 11.49, *P* < 0.0001]. **P < 0.01 and ***P < 0.001 vs 1 G; †††*P* < 0.001 vs HT.

Baseline body weight did not differ significantly among the 1 G, NHT, and HT groups (22.0 ± 1.3, 20.5 ± 1.2, and 23.7 ± 0.9 g, respectively; Table 1). Body weight remained significantly lower in both BVL groups than in the sham group throughout the experimental period, with no significant difference between the HT and NHT groups (Fig. 2A). Similarly, BVL markedly impaired the righting reflex, as reflected by prolonged righting time (Fig. 2B) and increased righting distance (Fig. 2C). These deficits persisted through day 10 and were not improved by repeated HT.

**Table 1:** Baseline characteristics of the experimental groups.

Parameter	1 G	NHT	HT
Body weight (g)	22.0 ± 1.3	20.5 ± 1.2	23.7 ± 0.9

**Fig. 3:**
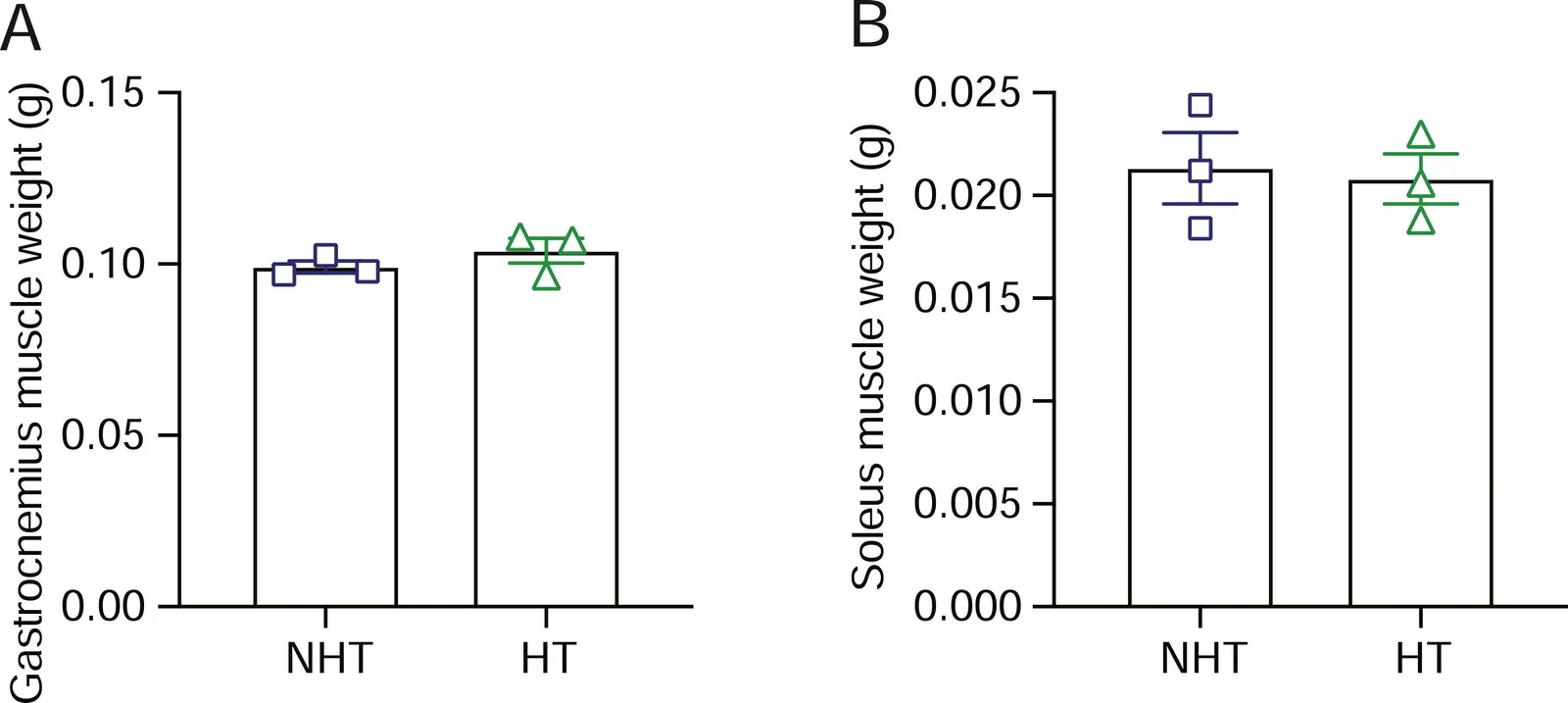
Skeletal muscle weights did not differ between the HT and NHT groups following bilateral vestibular lesion. (A) Gastrocnemius muscle weight and (B) soleus muscle weight in the hypergravity training (HT) and no hypergravity training (NHT) groups on Day 7 after bilateral vestibular lesion (BVL) (n = 3 per group). Data are presented as individual values and mean ± SEM. Statistical comparisons between the HT and NHT groups were performed using an unpaired two-tailed Student’s *t*-test.

In contrast, rotarod performance was significantly affected by HT (Fig. 2D). Both BVL groups exhibited marked impairment on day 3 compared with the sham group. However, on Day 7, mice receiving repeated HT showed a significantly better rotarod performance than the NHT group, whereas the NHT group remained markedly impaired. By day 10, rotarod performance had recovered to a level comparable to that of the sham group in both BVL groups. In contrast, gastrocnemius and soleus muscle weights did not differ significantly between the HT and NHT groups (Fig. 3A, B).

### Repeated hypergravity training induces transcriptional remodeling in the dorsal root ganglia

**Fig. 4:**
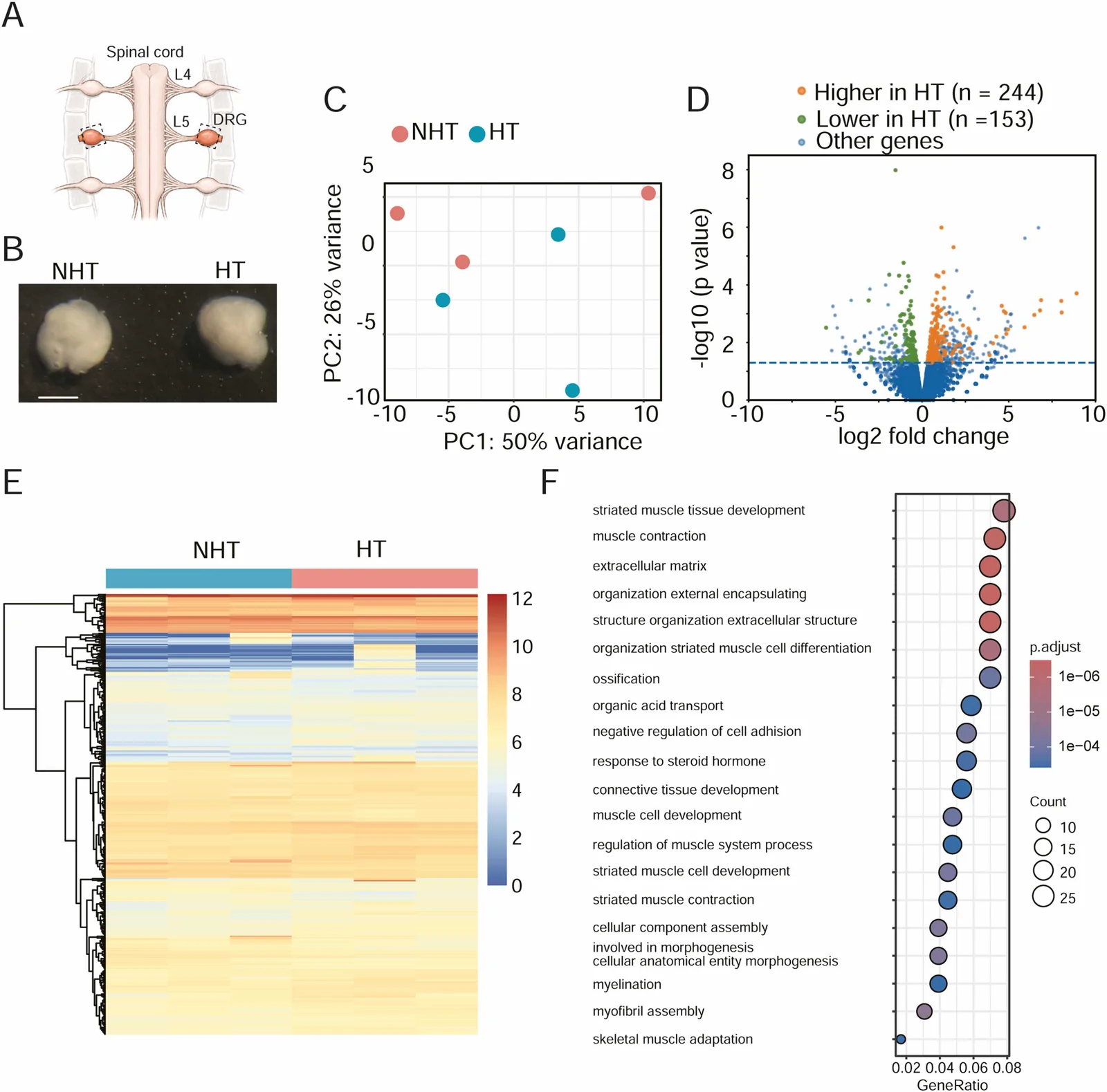
Repeated hypergravity training induces transcriptional remodeling in the dorsal root ganglia following bilateral vestibular lesion. (A) Schematic illustration of the anatomical location of the L5 dorsal root ganglia (DRGs) collected for RNA-sequencing analysis. (B) Representative photographs of L5 DRGs from the hypergravity training (HT) and no hypergravity training (NHT) groups before RNA extraction. No obvious gross morphological differences were observed between the groups. Scale bar = 1 mm. (C) Principal component analysis (PCA) of RNA sequencing data obtained from L5 dorsal root ganglia (DRGs) of the hypergravity training (HT) and no hypergravity training (NHT) groups on day 7 after bilateral vestibular lesion (BVL). PC1 and PC2 accounted for 50% and 26% of the total variance, respectively. (D) Volcano plot illustrating the distribution of gene-expression differences between the HT and NHT groups. (E) Heatmap of the 397 candidate genes identified using the exploratory criteria of raw P < 0.05 and an absolute difference in normalized expression values ≥ 20. (F) Gene Ontology (GO) enrichment analysis of the 397 candidate genes. GO terms with a Benjamini–Hochberg-adjusted P value < 0.05 were considered significantly enriched.

No obvious gross morphological differences were observed in the L5 DRGs between the HT and NHT groups before RNA extraction (Fig. 4B). RNA sequencing was performed using L5 DRGs collected from the HT and NHT groups on day 7 after BVL. Representative neuronal and sensory neuron markers expressed in the DRG, including Tubb3, Snap25, Scn9a, Avil, and Prph, were robustly detected across all samples. In contrast, canonical myogenic regulatory genes, including Myod1, Myog, and Pax7, were absent or detected only at negligible levels. A total of 49,585 annotated genes were analyzed (Supplementary Table 1). Following total count and quantile normalization, 397 candidate genes meeting the exploratory criteria (raw P < 0.05 and an absolute difference in normalized expression values ≥ 20) were identified between the HT and NHT groups (Supplementary Table 2). Of these, 244 genes were upregulated and 153 genes were downregulated in the HT group compared with the NHT group (Fig. 4D). Principal component analysis showed a tendency toward separation between the two groups, suggesting differences in their overall transcriptomic profiles (Fig. 4C). Hierarchical clustering further demonstrated distinct gene expression patterns between the HT and NHT groups (Fig. 4E).

GO enrichment analysis indicated significant enrichment of muscle-related biological processes, including muscle contraction and striated muscle development. In addition, processes related to myelination, axon ensheathment, and glial cell differentiation were significantly enriched. Myelination was included among the top 20 GO terms shown in Fig. 4F, whereas axon ensheathment and glial cell differentiation were also significantly enriched but were not included among the top 20 terms displayed.

## Discussion

The present study demonstrates that repeated HT selectively improved motor coordination following BVL without restoring vestibular-dependent righting reflexes. Although body weight loss and impairment of the righting reflex persisted throughout the experimental period, HT resulted in significantly better rotarod performance on day 7 compared with NHT. Furthermore, RNA sequencing of the L5 DRGs revealed transcriptional differences associated with repeated HT. These findings suggest that somatosensory adaptations may contribute to the improved motor performance observed after repeated HT, rather than restoration of vestibular function itself.

The differential effects of HT on the righting reflex and rotarod performance provide important insight into the mechanisms underlying vestibular compensation. The righting reflex primarily depends on vestibular and visual inputs and therefore directly reflects vestibular function, whereas successful rotarod performance additionally requires proprioceptive feedback, coordinated limb movement, cerebellar motor learning, and spinal motor control [14]. A previous study has shown that rotarod performance is improved by complex motor training involving the limbs and that the addition of somatosensory inputs further enhances motor recovery [15], [16]. Thus, the selective improvement in rotarod performance observed in the HT group suggests that repeated HT promoted compensatory motor adaptation rather than restoration of vestibular-dependent reflex pathways, consistent with the concept of sensory reweighting.

Our previous studies demonstrated that prolonged hypergravity attenuates the gain of the vestibulo-cardiovascular reflex by reducing vestibular afferent activity [5], [8], [17], [18] and that this deficit can be restored by repetitive vestibular electrical stimulation [9]. Although vestibular electrical stimulation and HT represent distinct forms of sensory stimulation, both interventions were associated with improved functional outcomes despite acting through different sensory modalities. Together with the present findings, these results suggest that vestibular compensation is not restricted to a specific type of sensory stimulation. Rather, repeated sensory inputs may engage activity-dependent plasticity within vestibular-related neural circuits. These findings support the concept that the magnitude and persistence of sensory input, rather than its modality, may be key determinants of adaptive vestibular plasticity.

RNA-sequencing analysis identified 397 candidate genes between the HT and NHT groups using exploratory criteria. Representative neuronal and sensory neuron markers were robustly expressed, whereas canonical myogenic regulatory genes, including *Myod1, Myog,* and *Pax7*, were absent or detected only at negligible levels, arguing against a canonical myogenic differentiation program within the DRG. However, only five genes met stringent criteria incorporating multiple-testing correction (FDR < 0.05 and absolute fold change > 2). Therefore, the transcriptomic and GO analyses should be regarded as exploratory and hypothesis-generating rather than definitive evidence of specific molecular pathways.

Muscle-related biological processes remained enriched in the revised GO analysis, although gastrocnemius and soleus muscle weights did not differ between the HT and NHT groups. Notably, myelination, axon ensheathment, and glial cell differentiation were also enriched, with several associated genes, including *Mbp, Mpz, Sox10, Pmp2,* and *Gjb1* [19]. These findings suggest that repeated HT may induce peripheral glial and myelination-related adaptations that influence somatosensory signal transmission. However, this interpretation remains hypothetical because myelin structure and nerve conduction were not directly assessed. Another limitation is that behavioral assessments were not performed during the first 2 days after BVL; therefore, we cannot determine whether HT attenuated the initial motor impairment or facilitated subsequent recovery.

In conclusion, repeated HT selectively improved motor coordination after BVL without restoring vestibular-dependent righting reflexes. This functional improvement was accompanied by transcriptional differences in the DRGs including enrichment of muscle-related and myelination-related biological processes. Together with our previous studies demonstrating the beneficial effects of vestibular electrical stimulation [9], the present findings support the possibility that repeated sensory stimulation, regardless of its modality, promotes activity-dependent vestibular plasticity when delivered with sufficient intensity and duration. These findings provide new insight into the mechanisms underlying vestibular compensation and may contribute to the development of sensory stimulation-based rehabilitation strategies for vestibular disorders.

## Authors' contributions

Chikara Abe was responsible for study planning. Yuta Masuda, Kakeru Shimizu, Marina Khatun, Risa Shimane and Nanaka Tameike conducted the experiments. All authors checked and revised the manuscript.

## CRediT authorship contribution statement

**Yuta Masuda:** Writing – review & editing, Methodology, Investigation, Funding acquisition, Formal analysis, Data curation. **Chikara Abe:** Writing – review & editing, Writing – original draft, Validation, Supervision, Project administration, Funding acquisition, Conceptualization. **Yukari Takeda:** Writing – review & editing. **Nanaka Tameike:** Investigation. **Risa Shimane:** Investigation. **Sajjad Hossain:** Writing – review & editing. **Marina Khatun:** Writing – review & editing. **Kakeru Shimizu:** Investigation.

## Ethics declaration

This study was conducted in accordance with the ARRIVE (Animal Research: Reporting of In Vivo Experiments) guidelines. The experimental protocols were approved by the Animal Research Committees of the University of Fukui (R08012).

## Ethics approval

All animal procedures were conducted in accordance with the Guiding Principles for the Care and Use of Animals in the Field of Physiological Science established by the Physiological Society of Japan. The experimental protocols were approved by the Animal Research Committees of the University of Fukui (R08012).

## Consent for publication

Not applicable.

## Funding

This study was supported by the Japan Society for the Promotion of Science Grant-in-Aid for Scientific Research (B) (26K02750 to C.A.), the Japan Society for the Promotion of Science Grant-in-Aid for Challenging Exploratory Research (25K22774 to C.A.), the Japan Society for the Promotion of Science Grant-in-Aid for Transformative Research Areas (A) (Publicly Offered Research) (26H01852 to C.A.), the Japan Society for the Promotion of Science Grant-in-Aid for Early-Career Scientists (26K18556 to Y.M.) and Cabinet Office Grant/Funding for Innovation Environment Enhancement Program (CA).

## Declaration of Competing Interest

The authors declare that they have no known competing financial interests or personal relationships that could have appeared to influence the work reported in this paper.
